# Study on the Absorption Characteristics and Laser Damage Properties of Fused Silica Optics under Flexible Polishing and Shallow DCE Process

**DOI:** 10.3390/mi12101226

**Published:** 2021-10-08

**Authors:** Wanli Zhang, Feng Shi, Ci Song, Ye Tian, Yongxiang Shen

**Affiliations:** Laboratory of Science and Technology on Integrated Logistics Support, College of Intelligence Science and Technology, National University of Defense Technology, Changsha 410073, China; zhangwanli17@nudt.edu.cn (W.Z.); songci@nudt.edu.cn (C.S.); tianyecomeon@sina.cn (Y.T.); xiangyueni@163.com (Y.S.)

**Keywords:** fused silica, laser induced damage threshold, magnetorheological finishing, ion beam finishing, dynamic chemical etching

## Abstract

The enhancement of laser damage resistance of fused silica optics was a hotspot in scientific research. At present, a variety of modern processes have been produced to improve the laser induced damage threshold (LIDT) of fused silica optics. They included pre-treatment processes represented by flexible computer controlled optical surfacing (CCOS), magnetorheological finishing (MRF), ion beam finishing (IBF), and post-treatment processes represented by dynamic chemical etching (DCE). These have achieved remarkable results. However, there are still some problems that need to be solved urgently, such as excessive material removal, surface accuracy fluctuation in the DCE process, and the pollution in MRF process, etc. In view of above problems, an MRF, CCOS, IBF and shallow DCE combined technique was used to process fused silica optics. The surface morphology could be greatly controlled and chemical etching depth was reduced, while the LIDT increased steadily. After processing by this combined technique, the LIDT increased to 12.1 J/cm^2^ and the laser damage resistance properties of fused silica were significantly enhanced. In general, the MRF, IBF, CCOS and shallow DCE combined technique brought much help to the enhancement of laser damage resistance of fused silica, and could be used as a process route in the manufacturing process of fused silica.

## 1. Introduction

As a material with excellent optical properties, fused silica was widely used in the manufacturing process of key optics in high-energy laser systems, such as National Ignition Facility (NIF) in the United States, SG-III laser facility in China and Laser Megajoule system in France, etc. [1,2,3]. For these laser systems, their service performance and life were limited by the quality and laser damage characteristics of fused silica optics. After years of research, pre-treatment processes including reactive ion etching (RIE) and post-treatment processes including dynamic chemical etching (DCE) had already been developed by scholars, which were applied to manufacture fused silica optics and achieved good results. Sun Laxi found that RIE technology could significantly improve the laser induced damage threshold (LIDT) of fused silica optics [4]. Under the conditions of 355 nm wavelength and a single longitudinal mode with about 3 ns (full wave at half maximum, FWHM), the 0 probability damage threshold could reach to 7.5 J/cm^2^ after the RIE process. J. Bude found particle pollution on the surface limited the LIDT improvement of fused silica optics. After advanced migration process (AMP) 3.0 process, the particles were obviously restricted, and the surface was free from damage under 351 nm, 3 ns (FWHM), 9.5 J/cm^2^ laser irradiation [5]. Through the modern process technique, the LIDT of fused silica had been greatly improved.

Even so, the laser damage resistance of fused silica optics still could not satisfy the service requirements, so scholars considered that the existence of micro defects limited the further improvement of LIDT of fused silica optics, including small-scale mechanical defects in the sub-surface damage (SSD) layer [6,7], contaminated elements [8,9], micro chemical structure defects [10], etc. These micro defects would affect the laser beam transmission or cause excessive energy deposition, and finally induce laser damage.

Scholars decided to examine the micro defects issue further. Shao Ting found that a deep DCE technique could remove chemical structure defects (oxygen deficit center (ODC)/non-bridging oxygen hole center (NBOHC) formed in RIE process [11]. Sun Laixi also found that RIE combined with deep DCE technique could effectively remove kinds of micro defects [12]. After removing several microns of materials, not only the Ce element introduced by traditional grinding process was removed, but also the impurity ions introduced in RIE process were obviously controlled. Through RIE and deep DCE process, the LIDT of the optics was significantly improved. It should be noted that the material removal amount in the DCE post-treatment process was basically in the order of microns. The reasons were: (1) the pollution layer introduced by previous process was thick; (2) a larger removal depth was conducive to the exposure of purer substrate and the LIDT of optics was improved, which was beneficial to the service life of laser systems. However, deep DCE etching was often accompanied by deterioration of surface morphology and enrichment of SiF_6_^2−^, which was not conducive improving the damage threshold further [13,14,15,16]. Therefore, we needed to explore a new process route to increase the LIDT of fused silica optics while reducing the etching depth in the DCE process.

Ion beam finishing (IBF) was a non-contact flexible technique which could achieve material removal by high-energy ion beam [17]. During the IBF process, the impurities could be effectively removed and would not introduce new damage [18]. The LIDT of the optics could reach up to 10.5 J/cm^2^ [19]. Magnetorheological finishing (MRF) was a flexible polishing technique with the advantages of high efficiency, high surface accuracy and low sub-surface damage, which had been used in the pre-treatment process of fused silica optics [20]. However, the damage density of optics without the chemical etching process was still large under laser irradiation of 8 J/cm^2^, which was related to the residual metal elements such as Fe. The existence of metallic pollution would increase the absorption of laser energy and result in laser damage [21]. For the DCE technique with deep etching depth, we had already realized that it could remove the pollution and improve LIDT of the optics. However, the shortcomings of deep etching also should be considered. In terms of the changes of fused silica with a small amount material removal in DCE process, few studies had been done. For these reasons, the effect of the MRF, IBE, shallow DCE technique in the threshold lifting process of fused silica optics was worth being researched further.

In this work, the MRF, IBF, computer controlled optical surfacing (CCOS) (this technique was used to control the medium-high frequency errors introduced in MRF process), shallow DCE technique was used to fabricate fused silica optics, and the changes of surface morphology, photo-thermal absorption, and LIDT were explored. In Section 2, sample preparation, the manufacturing technique and measurement were recommended. The test results are discussed in Section 3. In Section 4, we discuss the results in Section 3, and the relative phenomena are explained. The conclusion is represented in Section 5.

## 2. Materials and Methods

### 2.1. Sample Preparation

We had prepared four square optics (type: Heraeus 312 (Likabao Co. Ltd., Chengdu, China), serial number: 1–4, damage layer depth: 1–2 μm) with the size of 35 mm × 35 mm × 10 mm, produced by the same vendor with conventional grinding and polishing process.

Pre-treatment process: Sample 1 was blank and used as control. Sample 2 was processed by MRF (Detailed parameters was listed in Table 1). Sample 3 was processed by MRF, CCOS combined technique (Detailed parameters was listed in Table 1 and Table 2). Sample 4 was processed by MRF, CCOS, IBF (Detailed parameters was listed in Table 1, Table 2 and Table 3).

Post-treatment process: After pre-treatment processing, sample 1–4 was processed by the DCE technique including inorganic acid cleaning process and hydrogen fluoride (HF)-based etching process. The DCE process was carried out under megasonic conditions which were produced by a Teflon-lined, multi-frequency megasonic transducer (MultiMEGt 430 kHz (Changsha, China), 1.3 MHz), and the whole DCE process was divided into four steps: (1) inorganic acid (70 wt.% HNO_3_ and 40 wt.% H_2_O_2_, volume ratio: 2:1) (Sinopharm Chemical Reagent Co., Ltd., Shanghai, China) was used for pre-cleaning (time: 80 min), to remove the pollution on the surface introduced by pre-treatment and transportation process; (2) deionized water (Laser fusion research center, Mianyang, China) was used to remove the residual inorganic acid, after which the samples were dried by air; (3) Etchant (49 wt.% HF and 30 wt.% NH_4_F, volume ratio: 1:4) (Sinopharm Chemical Reagent Co., Ltd., Shanghai, China) was used to etch the surface materials and the etching rate was determined to be 0.1 μm/min. Sample 1–4 was etched for 1 min; (4) after the HF-based etching process, the samples were cleaned by deionized water. Steps 1–4 were carried out in a Class 100 clean room.

### 2.2. Test and Characterization

#### 2.2.1. Power Spectral Density Analysis

In this work, the power spectral density (PSD) analysis method was carried out, and the valid region of the PSD curve was chosen to analyze the changes in manufacturing process. The PSD curve showed the correlation between amplitude and specific frequency, and could be used for the medium-high frequency error analysis of the optical surface.

#### 2.2.2. Photo-Thermal Absorption Test

The absorptivity of optics was detected by a large aperture photo-thermal absorption platform (ZC Co., Ltd., Hefei, China). When the surface was irradiated by laser, some areas absorbed energy, and resulted in a rise in temperature, which would cause the changes of refractive index or other parameters. This minor difference would be captured by the detector, and then the absorptivity could be calculated. During the test, the inspection caliber was set at 35 mm × 35 mm, step length 1 mm, pump power 2 W, pulse repetition frequency (PRF) 50 kHz, polarimetric whitening filter (PWF) 950, integral time 300 ms, test wavelength 355 nm. The measurement method was transmission and the test sensitivity was better than 0.05 ppm.

#### 2.2.3. Laser Induced Damage Threshold Test

The LIDT test was carried out with 1-on-1 mode on the LIDT test platform equipped with a frequency-tripled Neodymium (Nd): Yttrium Aluminum Garnet (YAG) laser. The laser could produce 5 ns (FWHM) pulse in a single-longitudinal mode with the repetition rate of 1 Hz. The wavelength of the laser was operated at 355 nm and the spot diameter of laser beam was about 1.2 mm. During the test, ten testing sites with a certain laser flux were chosen on the surface and the distance between the two sites was 3 mm. The laser damage spot was observed by a long-focal-distance camera. The damage probability was obtained by calculating the number of the laser damage spot at each gradient.

#### 2.2.4. Damage Morphology Test

The damage morphology was detected by high-resolution microscope (model: VHX-600E, Keyence Co., Ltd., Osaka, Japan). The magnification was operated at 500 times. Through the test, the morphology characteristics of laser damage could be obtained.

#### 2.2.5. Roughness Test

The roughness of sample 1–4 after pre-treatment technique was detected by MicroProf WLI (Zygo Co., Ltd., California, CA, USA). Nine spots were chosen and a single region was set at 0.47 mm × 0.35 mm. The roughness was taken from the average value of the nine spots.

## 3. Results

### 3.1. PSD Valid Region Results

To accurately grasp the changes of the medium-high frequency of optical surface, we extracted the PSD information of sample 3–4, and the valid region was chosen to be analyzed.

**Figure 1:**
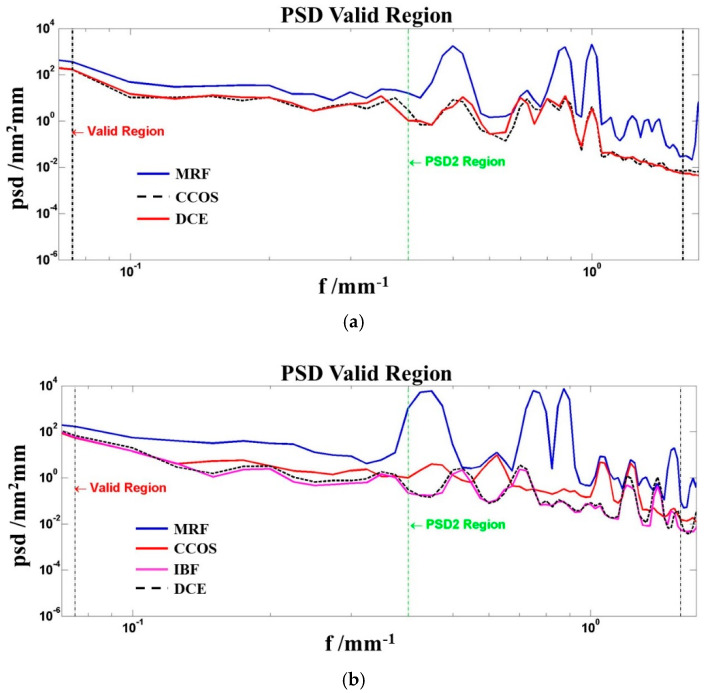
Power spectral density (PSD) results of samples: (**a**) 3–PSD information; (**b**) 4–PSD information.

In Figure 1a, it can be seen that specific medium-high frequency errors were introduced in the MRF process. After the CCOS process, the error amplitude significantly decreased and the PSD curve remained unchanged after the shallow DCE process. Compared with Figure 1b, the medium-high frequency errors were further controlled after the IBF process and the effect of the DCE kept stable, which indicated that the IBF, CCOS technique did well in terms of the frequency errors control. The shallow DCE process would not affect the amplitude of frequency errors.

### 3.2. Photo-Thermal Weak Absorption

In order to clarify the absorption characteristics, a large aperture photo-thermal absorption platform was used to detect the absorptivity level of sample 1–4 (processed by pre-treatment technique).

**Figure 2:**
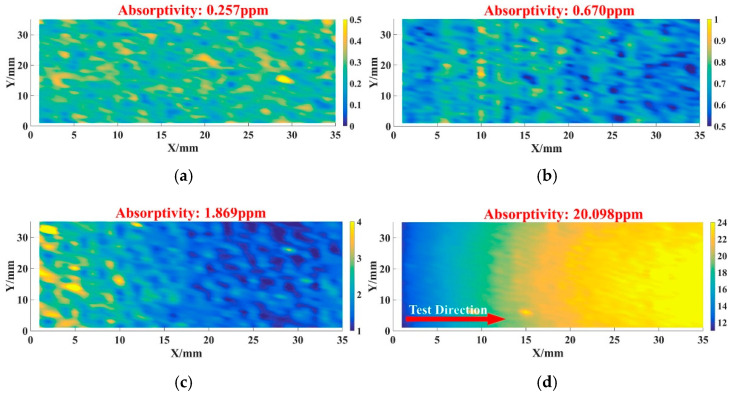
Absorptivity result of samples before dynamic chemical etching (DCE) process: (**a**) 1 absorptivity result; (**b**) 2 absorptivity result; (**c**) 3 absorptivity result; (**d**) 4 absorptivity result.

In Figure 2, the absorption of sample 1–3 was 0.257 ppm, 0.670 ppm and 1.869 ppm, respectively. As for sample 4, its absorptivity was relatively high and came to 20.098 ppm compared with the other samples. It could also be seen that the absorption figure (Figure 2d) of sample 4 showed an obvious transition trend, which might be related to the IBF technique itself. The difference of absorption between the samples needed to be investigated further.

After that, we detected the absorption level of sample 1–4 after the DCE post-treatment process.

**Figure 3:**
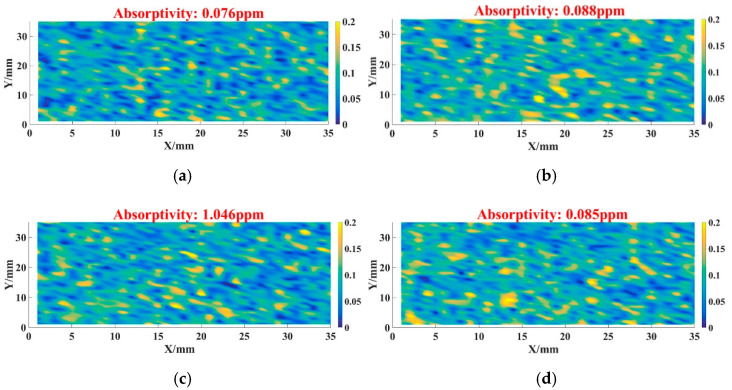
Absorptivity result of samples after DCE process: (**a**) 1—absorptivity result; (**b**) 2—absorptivity result; (**c**) 3—absorptivity result; (**d**) 4—absorptivity result.

According to Figure 3, the absorptivity of unprocessed sample 1 was 0.076 ppm. For sample 2, its absorptivity was 0.088 ppm, which was slightly higher than that of sample 1. The absorptivity of sample 3 was 1.046 ppm, and the absorptivity level was higher than sample 1–2. In Figure 2, the absorptivity of sample 3 was also higher than that of sample 1–2. We considered that the increasing of absorptivity related to CCOS technique, and the specific factors were discussed in Section 4. For sample 4, its absorptivity value came to 0.085 ppm after processing, and its absorption characteristics were significantly improved.

### 3.3. Laser Induced Damage Test

**Figure 4:**
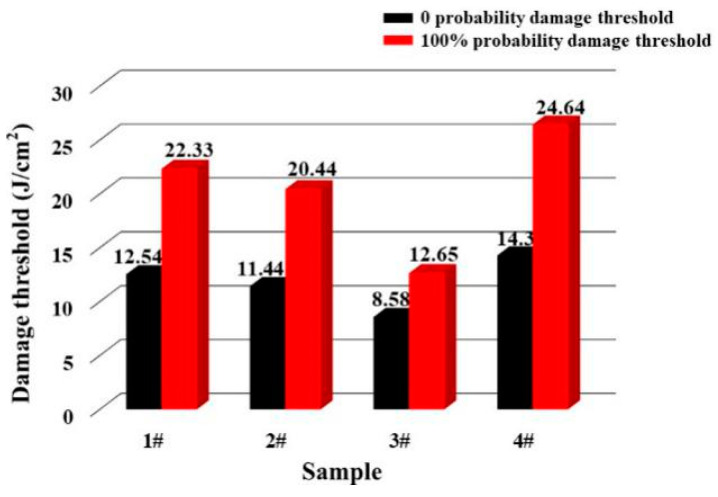
Results of laser damage threshold.

In order to explore the changes of anti-laser damage characteristics of optics under different techniques, an LIDT platform was used to detect the damage threshold of sample 1–4. The 0 probability damage threshold and 100% damage threshold results are shown in Figure 4.

In Figure 4, the 0 probability and 100% probability damage threshold of sample 4 were 14.3 J/cm^2^, and 24.64 J/cm^2^, respectively. Compared with other samples, its anti-laser damage characteristics were the best. The 0 probability and 100% probability damage threshold of sample 1 were 12.54 J/cm^2^ and 22.33 J/cm^2^, which were close to that of sample 2. For sample 3, its damage threshold was the lowest, and the values were 8.58 J/cm^2^ and 12.65 J/cm^2^, respectively. Through the results, it was determined that the trend of 0 probability damage threshold of different samples was the same as that of the 100% probability damage threshold, namely 4 > 1 = 2 > 3.

**Figure 5:**
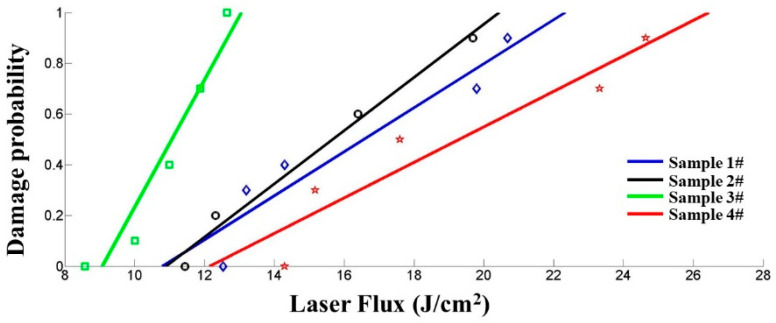
Fitting curves of laser damage threshold.

In order to clarify the difference of damage threshold between 1–4 further, we fitted the threshold test results. Figure 5 presents the correlation between laser damage probability and laser flux.

In Figure 5, sample 4 still had the highest LIDT, and the fitting value was 12.1 J/cm^2^. After processing by MRF, CCOS, IBF and the shallow DCE combined technique, the laser damage resistance of the optical surface was significantly improved. The fitting value of sample 2 was 10.9 J/cm^2^, which was a little higher than that of sample 1 (10.8 J/cm^2^). The fitting value of sample 3 was 9.1 J/cm^2^, which was lower than those of the other three samples.

From Figure 3, Figure 4 and Figure 5, it can be seen that the absorptivity and LIDT of samples 2–4 have a good correspondence between each other. When the surface was in a low-energy absorption state, it was easy for the optics to obtain a high laser damage threshold. Though sample 1 had the lowest absorption, its LIDT value was the same as sample 2, represented that there were other factors affecting its damage characteristics.

### 3.4. Laser Damage Morphology Results

**Figure 6:**
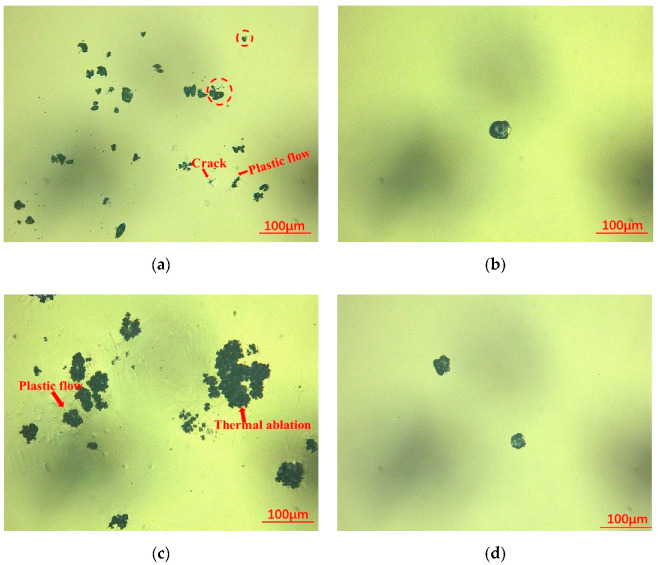
Laser damage morphology results: (**a**) 1—laser damage morphology; (**b**) 2—laser damage morphology; (**c**) 3—laser damage morphology; (**d**) 4—laser damage morphology.

In this part, we detected the laser damage morphology after LIDT test by high-resolution microscope, and the results are shown in Figure 6.

In Figure 6b,d, it can be seen that shallow shell or violet damage pits were dominant on the surface of sample 2 and 4, and the diameter of the damage pit was about 25–30 μm. For sample 1, many types of laser damage appeared on the surface, including matrix cracks and thermal abolition. At the same time, obvious thermoplastic flow could also be observed on the surface. The laser damage of sample 1 might link to the existence of sub-surface damage layer introduced in traditional polishing. For sample 3, the main laser damage was thermal ablation, plastic flow and content splashing, which could be observed at the damage area. Combined with Figure 2c and Figure 3c, we considered that the damage morphology was also related to the CCOS technique.

## 4. Discussion

As shown in the results in Section 3.2 and Section 3.3, it was easy to obtain a low-absorption surface by the MRF, CCOS, IBF and shallow DCE combined techniques, and the LIDT was much higher than others. In previous studies in our laboratory, we had already realized the advantages of MRF, IBF pre-treatment techniques [19,22]. For MRF techniques, high-efficiency material removal was achieved under the contacting between the flexible polishing ribbon and the optical surface, without introducing sub-surface damage. IBF was a non-contact manufacturing technique, and the material was removed by high-energy ion beams bombarding the optical surface. The technique would not introduce absorptive pollutants during the process. The IBF technique could effectively peel the hydrolysis layer and unstable layer, restrict the damage precursors and improve the anti-laser damage characteristics [17]. In sum, the MRF and IBF technique itself could provide a certain amount of help for improved performance of fused silica optics.

Though many scholars had already confirmed that MRF and IBF improved the surface quality of fused silica optics, a few questions were still worth discussing in this work, such as (1) sample 1 had lower absorptivity than sample 4, but its LIDT was lower than sample 4. What were the reasons that led to mismatching between photo-thermal absorption and LIDT; (2) after processing by the CCOS technique, the medium-high frequency errors of sample 3 were controlled (Figure 1a), but its damage threshold decreased compared with the other three samples. This phenomenon was not in line with expectations; (3) the IBF technique had been shown to improve the laser damage resistance of the optics in Section 3. However, the absorption of sample 4 (Figure 2d) after the IBF process was relatively higher than that of the other samples, which might not be in line with the prospective results.

For question 1, the sub-surface damage layer was thought to be the major factor, its existence led to the mismatching between photo-thermal absorption and LIDT. In the traditional polishing process, material removal was mainly achieved by a scratching effect between the water-based abrasive and the optical surface, and a sub-surface damage layer with a certain depth about hundreds nanometers to a few microns would form under the surface. In the sub-surface damage layer, a small amount of impurities and scratches existed, and they would cause intense energy absorption in the LIDT test, which would cause laser-induced damage.

After the traditional polishing process, no obvious defects appeared on the near surface. In a photo-thermal test, the relatively smooth near surface would not cause excess energy absorption when irradiated by the low-power pump of the absorption platform and the absorptivity would remain at a lower level. In contrast, the negative effects of the sub-surface damage layer were prominent in the LIDT test; the defects in the damaged layer would strongly absorb the laser energy and cause laser damage. In Figure 6, we found some clues from the damage morphology. It can be seen that various kinds of damage appeared on the surface of sample 1, including matrix cracks caused by thermal stress and thermal abolition caused by energy absorption. For the matrix cracks, it was related to the property of the materials changing in the micro area, while thermal ablation was related to the energy absorption mainly caused by impurities. All the laser damage on the surface was inseparable from the existence of the sub-surface damage layer. On the contrary, the damage to samples 2 and 4 was less, and mainly formed by the shallow shell, which reflected the effects of the sub-surface damage layer of sample 1 from the side.

For question 2, in a previous study [23], we had analyzed the medium-high frequency errors in detail and verified the correlation between medium-high frequency errors and substrate absorption. Lower medium-high frequency error amplitude could obtain lower absorption and reduce the laser damage probability. It was also clear that shallow DCE process would not affect the medium-high frequency errors. However, the conclusion was not applicable to solve question 2. Based on the above description, we believed that the LIDT and absorption was not only driven by medium-high frequency errors.

In order to explore other factors further, we detected the roughness of sample 1–4 after the pre-treatment process.

**Figure 7:**
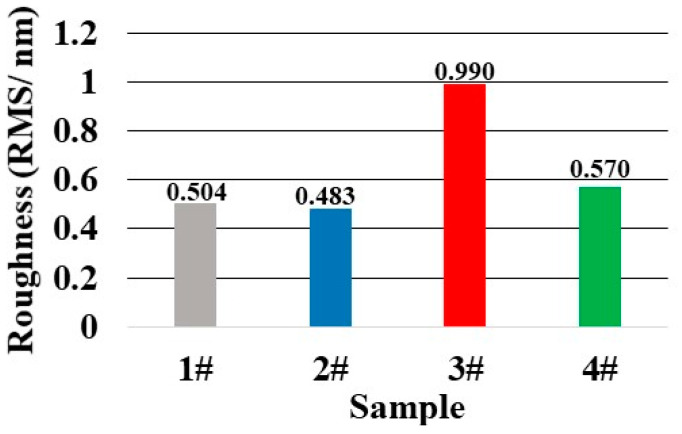
Roughness results of sample 1–4 after pre-treatment process.

According to Figure 7, it could be seen that the roughness (RMS) of sample 1 was 0.504 nm, and those of sample 2 and sample 4 were 0.483 nm and 0.570 nm, respectively. All the three samples had similar roughness values. On the contrary, the roughness of sample 3 increased to RMS 0.990 nm after the CCOS process, and the roughness deterioration might be a part of the reason for the decreasing of the LIDT. In the meantime, a certain depth of the hydrolysis layer would be introduced by the CCOS technique, which might also affect the LIDT to a certain degree.

For sample 3, the increasing of absorption (Figure 2c) also might be related to the hydrolysis layer introduced after the CCOS process. We considered that the hydrolysis layer could not be completely removed at the etching depth of 100 nm after the shallow DCE process, which led to its absorption being higher than other samples (Figure 3c). In Figure 6c, we could find the thermal ablation, plastic flow and content splashing caused by energy absorption, which was strongly related to the absorptive metallic element and other impurities that existed in hydrolysis layer.

Through the absorption results and damage morphology results, we verified the negative effect of the hydrolysis layer further. Due to the existence of the hydrolysis layer, not only did the photo-thermal absorption decrease, but the LIDT of sample 3 had also been affected. For question 2, we thought that the influence of medium-high frequency control was weaker than the disadvantages of roughness deterioration and the existence of hydrolysis together, which led to the LIDT decreasing in sample 3.

For question 3, the absorptivity fluctuation of sample 4 after the IBF process was related to the existence of a sputtering damage layer with different depth.

In the IBF process, high-energy ions would invade the surface for a certain distance driven by an ion beam. According to Lindhand Scharff and Schiott (LSS) range theory and Sigmund sputtering theory, the sputtering distance was related to ion beam energy:

(1) $$d(\varepsilon )=\frac{1-m}{2m}\gamma^{m-1}\frac{\varepsilon^{2m}}{NC_{m}}$$

*N* was the atomic density of material. *γ* was a mass related constant. *m* was a constant related to incident ion beam energy. *ε* was ion beam energy. *C_m_* was a constant related to interatomic potential. *d(ε)* was the sputtering distance.

In Equation (1), the ion sputtering distance showed a positive correlation with ion beam energy. During the ion beam sputtering process, lattice dislocation, holes and other micro structural defects would be introduced along with the invasion of ions. The structural defects would cause a small amount of energy absorption. To clarify the relation between sputtering distance and ion beam energy, we established a model [19].

(2) $$d_{s}=a(\cos\theta +\frac{3\sqrt{a_{\sigma }{}^{2}\sin^{2}\theta +a_{\mu }{}^{2}\cos^{2}\theta }}{a_{\mu }a_{\sigma }})$$

*a* was sputtering distance. *θ* was incident angle of ion beam. *a_σ_* and *a_μ_* were the deposition width of ion beam on the incident direction and perpendicular to the incident direction. *d_s_* was the sputtering damage layer depth. When the incident angle was 0 degree, Equation (2) could be changed to Equation (3):

(3) $$\begin{array}{l} d_{s}=a+a\frac{3\sqrt{a_{\mu }{}^{2}}}{a_{\sigma }a_{\mu }} \end{array}$$

According to Equations (1)–(3), the sputtering damage layer depth showed a positive correlation with the sputtering distance. When the energy beam increased, the ion beam sputtering distance increased and a deeper sputtering damage layer formed.

**Figure 8:**
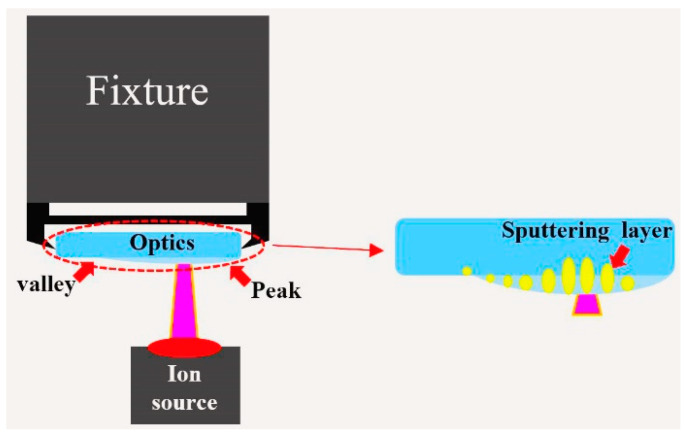
Sputtering damage layer formation process.

Due to the micro fluctuation of the surface, the ion beam energy in different areas was different, the principle of which is shown in Figure 8. The “peak” on the surface was closer to the ion source, its energy deposition was greater and would bring a deeper damage layer. On the contrary, the energy deposition on the “valley” was relatively low and the damage layer was relatively shallow.

**Figure 9:**
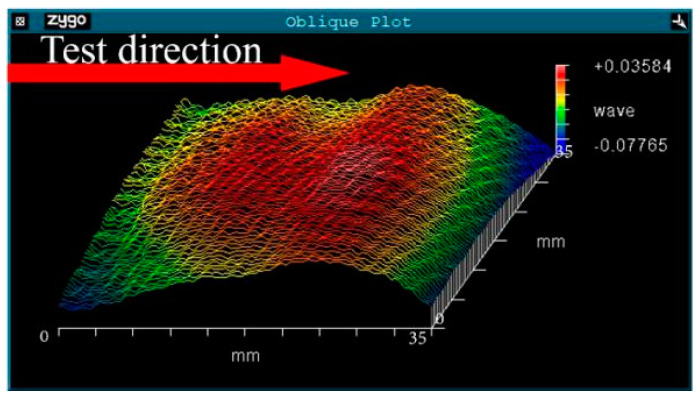
Surface shape test result.

In Figure 9, the surface shape test results of sample 4 were also basically consistent with the absorption result (Figure 2d), which confirmed our inference. From Equations (1)–(3) and the results shown in Figure 2d, Figure 8 and Figure 9, different ion beam energy would produce damage layers with different depth, and the existence of damage layers resulted in high absorption. It should be noted that the sputtering layer was tens of angstrom, and could easily be removed by the DCE process. So the sputtering damage layer would not affect the absorption and LIDT (Figure 3d and Figure 4).

In summary, the MRF, CCOS, IBF and shallow DCE combined techniques could remove the surface materials effectively along with keeping the surface quality stable, and the LIDT of fused silica optics could be improved steadily. Meanwhile, the sub-surface damage layer and the hydrolysis layer needed to be removed in order to inhibit the laser damage.

## 5. Conclusions

As an important optical material, fused silica had been applied to the manufacturing process of optics used in high-flux laser systems, and its manufacturing level had a vital influence on the performance of the laser systems. After years of effort, modern optical processing techniques represented by MRF, IBF, and DCE had been developed to manufacture fused silica and to improve its laser induced damage threshold. In this work, MRF, IBF, CCOS and the shallow DCE combined technique was chosen to process fused silica optics, and the changes of photo-thermal absorption and LIDT in the manufacturing process were also explored. After processing by combined technique, the photo-thermal absorption of the optic decreased to 0.085 ppm while LIDT increased to 12.1 J/cm^2^. From the results, it can be seen that absorption presents a strong correlation with LIDT, specifically that lower absorption could a obtain higher LIDT.

Meanwhile, we had found that some factors would affect the absorption properties and laser damage resistance of fused silica to varying degrees, including medium-high frequency errors, roughness, and the hydrolysis layer. Compared with other factors, the hydrolysis layer and the sub-surface damage layer had a great impact on LIDT. When irradiated by laser, the absorptive pollutants in the hydrolysis layer would cause complex laser damage. For the hydrolysis layer and the sub-surface damage layer, it could be removed by the MRF and IBF technique, which made the LIDT increase.

In conclusion, MRF, CCOS, IBF, and the shallow DCE combined technique could effectively control the photo-thermal absorption and improve the laser damage resistance as well as keeping the surface quality stable. This process could lay a foundation for the manufacturing process of fused silica optics with high laser damage resistance and ensure the development of laser systems.

## Figures and Tables

**Table 1:** Magnetorheological finishing (MRF) technique parameters of sample 2, 3, 4.

MRF Parameters	Value
Flow rate	130 L/h
Electric current	0.8 A
Wheel speed	240 rpm/min
Polishing abrasive	CeO_2_
Abrasive diameter	0.2 μm
Pressed depth	0.3 mm
Volumetric removal efficiency	3.6 × 10^7^ μm^3^/min
Removal depth	2.16 μm

**Table 2:** Computer controlled optical surfacing (CCOS) technique parameters of sample 3, 4.

CCOS Parameters	Value
Polishing abrasive	CeO_2_
Abrasive diameter	50 nm
Rotation speed	180 rpm/min
Polishing pad	Asphalt
Polishing pressure	0.9 kPa
Pad diameter	20 mm
Polishing time	1 h

**Table 3:** Ion beam finishing (IBF) technique parameters of sample 4.

IBF Parameters	Value
Gas	Argon
Incident angle	0°
Ion beam energy	900 eV
Vacuum chamber pressure	1 × 10^−4^ Pa
Material removal rate	11.5 × 10^−3^ μm^3^/min
Removal depth	200 mm
